# Exploratory analysis of predictive models in the field of myelitis: a systematic review and meta-analysis

**DOI:** 10.3389/fimmu.2025.1669338

**Published:** 2025-10-02

**Authors:** Jingjie Huang, Yusheng Zhao, Ziyi Zhang, Yupei Cheng, Bangqi Wu

**Affiliations:** ^1^ Acupuncture and Tuina School, Chengdu University of Traditional Chinese Medicine, Chengdu, Sichuan, China; ^2^ National Clinical Research Center for Chinese Medicine Acupuncture and Moxibustion, First Teaching Hospital of Tianjin University of Traditional Chinese Medicine, Tianjin, China; ^3^ Nanyang Medical College, Nanyang, Henan, China; ^4^ Postgraduate School, Tianjin University of Traditional Chinese Medicine, Tianjin, China

**Keywords:** myelitis, diagnostic model, prediction model, performance evaluation, clinical applicability

## Abstract

**Background:**

There has been a significant increase in the number of diagnostic and predictive models for myelitis. These models aim to provide clinicians with more accurate diagnostic tools and predictive methods through advanced data analysis and machine learning techniques. However, despite the growing number of such models, their effectiveness in clinical practice and their quality and applicability in future research remain unclear.

**Objective:**

To conduct a comprehensive methodological assessment of existing literature concerning myelitis modeling methodologies.

**Methods:**

We queried PubMed, Web of Science, and Embase for publications through October 23, 2024. Extracted parameters covered: study design, data origin, outcome criteria, cohort size, predictors, modeling techniques, and validation metrics. Methodological quality was evaluated using the PROBAST instrument, assessing potential biases and clinical applicability.

**Results:**

Among the 11 included studies, six focused on predictive diagnostic models, while five were centered on prognostic models. Modeling approaches comprised: logistic regression (n=6), Cox regression (n=2), deep learning (n=1), joint modeling (n=1), and hybrid machine learning/scoring algorithms (n=1). Multivariable logistic regression was the most frequently employed modeling algorithm in the current field. The most commonly used predictors for training diagnostic or prognostic models in myelitis were sex (n=6) and age (n=4). PROBAST evaluation indicated: (1) High bias risk (n=6): primarily from suboptimal data sourcing and analytical reporting gaps; (2) Unclear risk (n=4): mainly due to non-transparent analytical workflows; (3) Low risk (n=1). Pooled AUC for eight validated models reached 0.83 (95%CI: 0.75–0.91), demonstrating robust discriminative capacity.

**Conclusion:**

Although existing models demonstrate good discrimination in predicting myelitis, according to the PROBAST criteria, only one study exhibited a low risk of bias; analysis of data accessibility indicated that the model from only one study was directly available for public use. Consequently, future research should prioritize the development of models with larger cohort sizes, rigorous methodological design, high reporting transparency, and validation through multicenter external studies, enabling direct clinical translation to enhance their application value in clinical practice and improve healthcare delivery.

**Systematic Review Registration:**

https://www.crd.york.ac.uk/prospero/, identifier CRD42024623714.

## Introduction

Myelitis is an inflammatory disease affecting the nervous system, primarily involving the gray and white matter regions of the spinal cord. The etiology of this condition is diverse, encompassing viral and bacterial infections, autoimmune diseases, and associations with certain systemic disorders. The clinical manifestations of myelitis vary depending on the affected spinal cord regions. Typical symptoms include limb weakness, neuropathic pain, sensory deficits, autonomic dysfunction, and varying degrees of motor impairment (1–3). The disease can present with acute onset and, in severe cases, may result in permanent neurological damage. Common types of myelitis include acute transverse myelitis (4), autoimmune myelitis—such as neuromyelitis optica (NMO) or neuromyelitis optica spectrum disorders (NMOSD) (5, 6), multiple sclerosis (MS) (7), and systemic lupus erythematosus (SLE) (8, 9), as well as infectious myelitis caused by pathogens like viruses, bacteria, or fungi (10–13). Among these, poliomyelitis and NMOSD have garnered more attention and extensive research. Studies suggest that various forms of myelitis are highly disabling diseases. Patients often endure significant disease burdens due to symptoms such as pain (14), which severely impacts their emotional well-being and quality of life (15–18).

Although the etiology of myelitis varies—ranging from immune-mediated mechanisms to infections—each cause requires distinct management strategies and yields different prognostic outcomes. Nonetheless, early and effective diagnosis, risk assessment, and outcome prediction are critical for timely interventions, preventing disease progression, and improving patient quality of life (19). As such, developing models for predicting disease progression, risk, and classification of myelitis has become a significant focus of current research. Despite expanding myelitis prognostic frameworks in recent years, their methodological rigor and clinical translatability remain unvalidated. This systematic review synthesizes existing predictive constructs for myelitis populations, generating evidence-based guidance for clinical deployment and research advancement.

## Methods

Study protocol registered on PROSPERO (CRD42024623714).

### Search strategy

Three biomedical repositories (PubMed, Web of Science, Embase) were systematically queried from inception through October 23, 2024, limited to English publications. The keywords used included terms such as “myelitis,” “prediction model,” “predictive factors,” “predictors,” “model,” and “scoring.” The complete search strategy is detailed in **Table 1**. Our systematic review employed the PICOTS framework per CHARMS guidelines (20), which standardizes review objectives, search methodology, and eligibility criteria (21). Additionally, we utilized the CHARMS-PROBAST integrated Excel template developed by B. M. Fernandez-Felix and colleagues to streamline evidence extraction and bias appraisal for clinical prediction models (22). Core systematic review components include:

**Table 1:** Search strategy table.

Databases	Search formula
Pubmed	(myelitis[Title/Abstract] OR “spinal cord inflammation”[Title/Abstract] OR “transverse myelitis”[Title/Abstract] OR “inflammatory myelopathy”[Title/Abstract]) AND (prediction[Title/Abstract] OR predict*[Title/Abstract] OR prognos*[Title/Abstract] OR probability[Title/Abstract] OR score*[Title/Abstract] OR independent*[Title/Abstract]) AND (model[Title/Abstract] OR models[Title/Abstract] OR regression[Title/Abstract] OR equation*[Title/Abstract] OR score*[Title/Abstract] OR probability[Title/Abstract])
Web of Science	TS=(myelitis OR “spinal cord inflammation” OR “transverse myelitis” OR “inflammatory myelopathy”) AND TS=(prediction OR predict* OR prognos* OR probability OR score* OR independent*) AND TS=(model OR models OR regression OR equation* OR score* OR probability)
Embase	(myelitis:ti,ab OR ‘spinal cord inflammation’:ti,ab OR ‘transverse myelitis’:ti,ab OR ‘inflammatory myelopathy’:ti,ab) AND (prediction:ti,ab OR predict*:ti,ab OR prognos*:ti,ab OR probability:ti,ab OR score*:ti,ab OR independent*:ti,ab) AND (model:ti,ab OR models:ti,ab OR regression:ti,ab OR equation*:ti,ab OR score*:ti,ab OR probability:ti,ab)

Population (P): Patients with any type of myelitis.
Intervention (I): Developed and published diagnostic or prognostic models (with ≥2 predictors).
Comparator (C): No competing models.
Outcome (O): Any outcomes related to myelitis.
Timing(T): Predict outcomes based on the basic information at admission, clinical features, clinical scoring scale results, imaging characteristics, and laboratory indicators, or changes in features during the follow-up period.
Setting (S): The intended use of diagnostic prediction models is to individually predict the type of myelitis at an early stage, promote early diagnosis, and improve early clinical outcomes; The intended use of risk prediction models is to individually predict the later progression and changes of myelitis, helping to implement preventive measures and avoid adverse events.

#### Inclusion criteria

1. Myelitis patient cohorts;

2. Observational designs;

3. Prognostic algorithm development;

4. Myelitis-related outcomes.

#### Exclusion criteria

1. Non-predictive model studies;

2. Non-English publications;

3. Unretrievable full texts despite author outreach.

### Study selection and screening

Two investigators (Huang JJ, Zhang ZZ) independently conducted selection. Initial title/abstract screening determined eligibility. Full-text articles underwent inclusion/exclusion assessment, with duplicate removal. Disagreements were resolved through tripartite consensus deliberation (Huang JJ, Zhang ZZ, Wu BQ).

### Data extraction

Two researchers independently evaluated search results. Full-text eligibility assessments and discrepancies were resolved through deliberation or third-reviewer arbitration.

Data were documented via standardized Excel template (22), categorized as:

1. Basic Information: authors, year, design, participants, data sources, sample size;
2. Model Information: feature selection methodologies, model derivation techniques, validation approaches, performance quantifiers, missing data handling, continuous covariate transformation, final predictors, and model presentation formats.

Data extraction was performed by one researcher, and all data were subsequently verified by another researcher to ensure consistency and accuracy, with necessary corrections made as needed.

### Quality assessment

Study bias risk and applicability were evaluated using the Prediction Model Risk of Bias Assessment Tool (PROBAST) (23). Two investigators (Huang JJ, Zhang ZZ) conducted independent appraisals. PROBAST provides a critical appraisal framework for prediction model studies (development/validation/updating), comprising 20 signaling queries across four domains: Participants, Predictors, Outcomes, Analysis. Each query accepts five responses: “Yes,” “Probably Yes,” “No,” “Probably No,” or “No Information.” Domain-level bias was deemed high-risk when ≥1 query received “No”/”Probably No.” Overall low bias risk required all domains achieving low-risk status.

### Data synthesis and statistical analysis

Validation model AUC values underwent meta-analysis in RStudio (https://posit.co/download/rstudio-desktop/). Heterogeneity was quantified via I² index and Cochran’s Q test, with 25%/50%/75% thresholds denoting low/moderate/high heterogeneity (24). Effect model selection (fixed/random) depended on heterogeneity magnitude. Publication bias was assessed using Egger’s test (P>0.05 suggesting minimal bias) (25).

## Results

### Study selection

**Figure 1:**
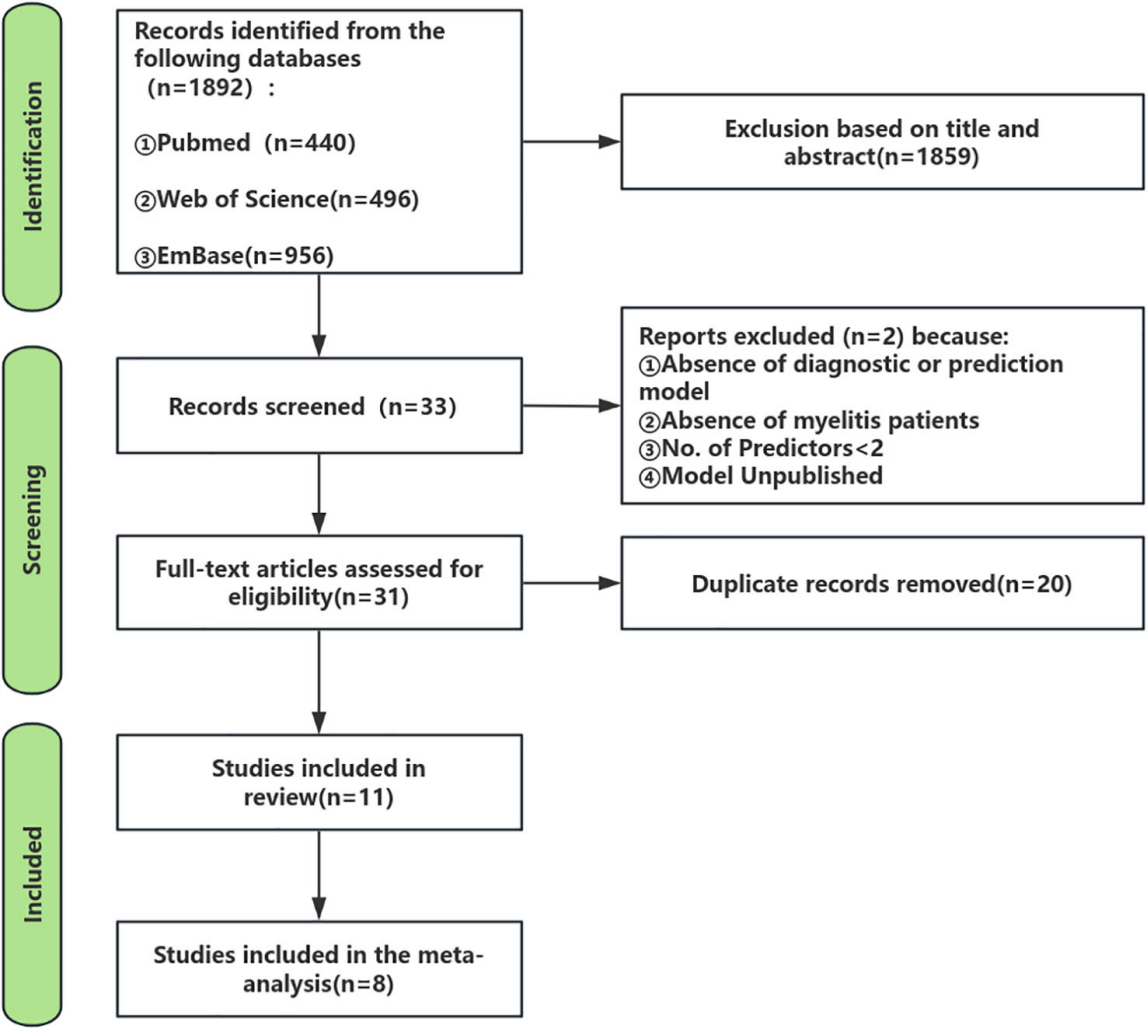
Evidence identification and screening methodology.

**Figure 1** delineates the systematic review/meta-analysis search flowchart, presenting comprehensive search methodology and outcomes.

As of October 23, 2024, the initial search across the three databases yielded a total of 1,892 records. In the first stage, screening titles and abstracts resulted in the selection of 33 articles that were relevant to the study topic (at this stage, articles that clearly did not develop prediction models or focused solely on risk factors were excluded, and some borderline articles proceeded to the next evaluation stage). In the second stage, applying the inclusion and exclusion criteria led to 31 articles being selected for further evaluation. During this evaluation phase, 2 studies were excluded after discussion and assessment because they did not constitute prediction models in the strict sense and had fewer than two predictors. In the third stage, after removing 20 duplicate records, the final review included 11 studies and 11 models.

### Study characteristics

Details regarding the literature, model names, and key algorithmic applications of the 11 included studies are summarized in **Table 2**; the study designs, validation methods, sample sizes, data processing, and comprehensive analytical techniques are detailed in **Table 3**. These studies, published between 2003 and 2024, include six multicenter collaborative studies, four single-center studies, and one study with unclear institutional reporting. Methodologically, the majority of studies (n=7) adopted a retrospective design, two incorporated both retrospective and prospective designs, and the remaining two consisted of one case-control study and one cross-sectional study. The study populations predominantly involved patients with neuromyelitis optica (NMO) (8 studies), three of which also included cohorts with multiple sclerosis (MS), and one included patients with myelin oligodendrocyte glycoprotein antibody-associated disease (MOGAD). The other studies focused on transverse myelitis (2 studies) and severe myelitis (1 study). The sample sizes across all studies ranged from 66 to 640 participants.

**Table 2:** Overview of literature information.

No. model	Author	Publication identifier (Title)	Publication journal	Model type	Model name	Algorithms
1	Fan Yang, 2024 (32)	Prediction of pulmonary infection in patients with severe myelitis by NPAR combined with spinal cord lesion segments	Frontiers in Neurology	Prognostic model	The nomogram model	Multivariate logistic Regression
2	Wenqin Luo, 2023 (33)	Visual disability in neuromyelitis optica spectrum disorders: prognostic prediction models	Frontiers in Immunology	Prognostic model	The nomogram model	LASSO regression and multivariate Cox regression
3	Lucia Campetella, 2023 (35)	A score that predicts aquaporin-4 IgG positivity in patients with longitudinally extensive transverse myelitis	European Journal of Neurology	Predictive diagnostic model	A score	Multivariate logistic regression
4	Liang Wang, 2022 (34)	Neuromyelitis Optica Spectrum Disorder With Anti-Aquaporin-4 Antibody: Outcome Prediction Models	Frontiers in Immunology	Prognostic model	The nomogram model	Extended Cox regression based on the Anderson-Gill (AG) model
5	Laura Cacciaguerra, 2022 (52)	Application of deep-learning to the seronegative side of the NMO spectrum	Journal of Neurology	Predictive diagnostic model	A DL algorithm	Deep learning convolutional neural network
6	Jingzi ZhangBao, 2021 (26)	Myelitis in inflammatory disorders associated with myelin oligodendrocyte glycoprotein antibody and aquaporin-4 antibody: A comparative study in Chinese Han patients	European Journal of Neurology	Predictive diagnostic model	A scoring model	Multivariate logistic regression
7	Laura Clarke, 2021 (27)	MRI Patterns Distinguish AQP4 Antibody Positive Neuromyelitis Optica Spectrum Disorder From Multiple Sclerosis	Frontiers in Neurology	Predictive diagnostic model	A prediction algorithm	Machine learning and scoring algorithms
8	Jacqueline Palace, 2019 (30)	Outcome prediction models in AQP4-IgG positive neuromyelitis optica spectrum disorders	Brain	Prognostic model	Joint modeling framework	Joint modeling approach
9	Laura Cacciaguerra, 2019 (31)	Brain and cord imaging features in neuromyelitis optica spectrum disorders	Annals of Neurology	Predictive diagnostic model	An algorithm	Multivariate logistic regression
10	Paula Barreras, 2018 (28)	Clinical biomarkers differentiate myelitis from vascular and other causes of myelopathy	Neurology	Predictive diagnostic model	A discriminatory model	Multivariate logistic regression
11	Dean M.Wingerchuk, 2003 (29)	Neuromyelitis optica Clinical predictors of a relapsing course and survival	Neurology	Prognostic model	Disease course models and survival models	Multivariate logistic regression and Cox regression

**Table 3:** Overview of basic data of the included studies.

Author, year	Study design	Type of disease	Study setting	Verification method	Sample size (training/test/validation)	Missing data handling	Sampling techniques	Cross-validation	Dimensionality reduction techniques	Hyperparameter adjustments
Fan Yang, 2024 (32)	Retrospective cohort	SM	Single-center	①Internal validation: Bootstrap; ②External validation: No	177/no/no	Direct exclusion	No	No	Univariate Analysis→Multivariate Logistic Regression	Not applicable
Wenqin Luo, 2023 (33)	Retrospective cohort	NMOSD	Single-center	①Internal validation: Bootstrap; ②External validation: No	640/no/no	Direct exclusion	Bootstrap Resampling	Yes, 10-fold cross-validation	LASSO regression	Yes
Lucia Campetella, 2023 (35)	Cohort combining retrospective and prospective approaches	LETM	Multi-center	①Internal validation: Yes; ②External validation: Completely independent	44/no/22	Unclean	No	No	Statistics-Driven Variable Screening	Not applicable
Liang Wang, 2022 (34)	Retrospective cohort	NMOSD	Multi-center	①Internal validation: Bootstrap; ②External validation: Completely independent	358/no/92	Unclean	No	No	Statistics-Driven Variable Screening	Not applicable
Laura Cacciaguerra, 2022 (52)	Retrospective (partially prospective) cohort study	NMOSD and MS	Multi-center	①Internal validation: Retain validation set; ②External validation: Yes	120/48/60	Unclean	Data Augmentation	No	Convolutionary Neural Network	Yes
Jingzi ZhangBao, 2021 (26)	Retrospective cohort	MOGAD and NMOSD	Single-center	①Internal validation: Unclean; ②External validation: No	255/no/no	Unclean	No	No	Stepwise procedure	Not applicable
Laura Clarke, 2021 (27)	Case-control	NMOSD and MS	Multi-center	①Internal validation: Bootstrap; ②External validation: No	166/no/no	Unclean	Case-control matching	No	Extensive Literature Review	Unclean
Jacqueline Palace, 2019 (30)	Retrospective cohort	NMOSD	Multi-center	①Internal validation: Bootstrap; ②External validation: No	441/no/no	Unclean	No	No	No	Not applicable
Laura Cacciaguerra, 2019 (31)	Cross-sectional study	NMOSD and MS	Multi-center	①Internal validation: Random split; ②External validation: No	120/no/61	Unclean	Stratified random sampling	No	No	No
Paula Barreras, 2018 (28)	Retrospective cohort	TM	Single-center	①Internal validation: Unclean; ②External validation: No	457/no/no	Direct exclusion	No	No	No	Not applicable
Dean M.Wingerchuk, 2003 (29)	Retrospective cohort	NMO	Unclean	①Internal validation: No; ②External validation: No	80/no/no	Unclean	No	No	No	Not applicable

TM, Longitudinally extensive transverse myelitis; NMOSD, Neuromyelitis optica spectrum disorder; MS, Multiple sclerosis; MOGAD, Myelin oligodendrocyte coprotein antibody (MOG-ab)-associated disease; NMO, Neuromyelitis optica; TM, Transverse myelitis; SM, Severe myelitis.

Among all the included studies, six focused on predictive diagnostic models, while five were prognostic models. Six studies utilized multivariate logistic regression algorithms to develop their models, and one employed Cox regression. Notably, Dean M. Wingerchuk et al. combined both multivariate logistic regression and Cox regression algorithms. Additionally, Wenqin Luo et al. integrated LASSO regression with Cox regression for modeling, Laura Cacciaguerra et al. (52) applied a deep learning convolutional neural network algorithm, Jacqueline Palace et al. adopted a joint modeling strategy, and Laura Clarke et al. utilized both machine learning and scoring algorithms in their modeling approaches.

The most frequently occurring predictor across the models was gender (featured in 6 models), followed by age (in 4 models). Other common predictors included number of relapses, central cord lesion, and long-segment transverse myelitis (LETM) (each appearing in 3 models). Predictors with relatively high frequency also included aquaporin-4 antibody (AQP4) serostatus (antibody titer), treatment duration, type of treatment regimen, spasticity, brainstem lesion, cervical cord lesion, and occurrence of optic neuritis (each present in 2 models).

### Models validation

Among the included studies, only three conducted both internal and external validation, while five performed internal validation only. Two studies did not carry out external validation, and their reporting on internal validation was unclear in the articles. Additionally, one study performed neither internal nor external validation (see **Table 3**).

### Technology application

Among the 11 included studies, the sample sizes used for training and testing the models are detailed in **Table 3**. Only three studies described their methods for handling missing data, all of which employed direct exclusion, while the remaining eight studies did not report any approach for addressing missing values. Just four studies utilized sampling techniques: Bootstrap Resampling, Data Augmentation, Case-control Matching, and Stratified Random Sampling were employed respectively. Only one study (Wenqin Luo et al. (33),) implemented 10-fold cross-validation.

Additionally, seven studies applied dimensionality reduction techniques: Lucia Campetella and Liang Wang both used Statistics-Driven Variable Screening; Wenqin Luo applied LASSO Regression; Laura Cacciaguerra et al. (52) utilized a Convolutional Neural Network for feature reduction; Jingzi ZhangBao adopted a Stepwise Procedure; Laura Clarke conducted an Extensive Literature Review to select variables; and Fan Yang first screened variables with p < 0.05 in univariate analysis before incorporating significant predictors into multivariate logistic regression for further selection.

Furthermore, only Wenqin Luo et al. and Laura Cacciaguerra et al. (52) explicitly reported the use of hyperparameter tuning in their studies, while Laura Clarke et al. did not provide clear information on whether hyperparameter optimization was performed.

### Performance metrics

Of the 11 studies included, the core performance metrics used and their performance evaluations are presented in **Table 4**. The model from Jingzi ZhangBao et al. demonstrated excellent discriminative ability (AUC 0.94), making it an outstanding diagnostic tool. The model developed by Laura Clarke et al. showed superior comprehensive performance (high precision and high F-score) and was significantly better than existing standards. The model by Fan Yang et al. had acceptable discriminative ability (AUC 0.77), but exhibited excellent calibration and clinical utility, making it a reliable prognostic risk prediction tool. In terms of methodological rigor and model performance, the models from Wenqin Luo et al. and the Laura Cacciaguerra (31) et al. are considered reliable, as they employed stringent internal validation and a comprehensive set of evaluation metrics. The respectable performance of earlier studies (e.g., Dean M.Wingerchuk et al.) should be interpreted with caution due to their higher risk of overfitting. The models by Laura Cacciaguerra (2022) et al. and Lucia Campetella et al. demonstrated exceptional discriminative ability (AUC > 0.9); the former is designed for classification tasks, and the latter for diagnostic purposes. The model from Liang Wang et al. showed moderate discriminative ability but excellent calibration and clinical utility, which is crucial for risk prediction models. The study by Jacqueline Palace et al. focused on interpreting risk factors rather than predicting individual outcomes; thus, its performance is reflected in the precision and significance of its effect estimates.

**Table 4:** Comprehensive analysis of performance metrics and model results.

Author, year	Assessment indicators	Model performance	Performance evaluation
Fan Yang, 2024 (32)	C-index	0.766	While its discrimination is not perfect, it demonstrates excellent calibration. Its clinical utility is confirmed by DCA, making it a reliable tool to support clinical decision-making.
	Calibration Plot	Highly consistent	
	Hosmer-Lemeshow	0.299	
	Decision Curve Analysis	Clinical net benefit	
Wenqin Luo, 2023 (33)	C-index	0.88-0.89	The model demonstrates excellent discrimination, highly accurate predicted probabilities, and has proven clinical utility across a wide range of decision thresholds.
	Calibration Plot	Highly consistent	
	Decision Curve Analysis	Clinical net benefit	
Lucia Campetella, 2023 (35)	Sensitivity	85%	The AIM score demonstrates exceptional diagnostic performance, with all key metrics being outstanding, making it a highly reliable and easy-to-use point-of-care tool.
	Specificity	95%	
	Likelihood Ratio	LR+ = 16.6 LR- = 0.2	
	AUC-ROC	0.93	
Liang Wang, 2022 (34)	C-index	0.66/0.65	It demonstrates only moderate discrimination, but exhibits superb calibration, and DCA confirms its clinical utility.
	Calibration Plot	Highly consistent	
	Decision Curve Analysis	Clinical Net Benefit	
Laura Cacciaguerra, 2022 (52)	Accuracy	0.97/0.95/0.93	It demonstrates excellent performance, with both accuracy and AUC exceeding 0.9, indicating that it can reliably distinguish between NMOSD and MS from brain MRI scans.
	AUC-ROC	0.93	
	False Positive Rate	0.04	
	Mean Absolute Error and Mean Squared Error		
Jingzi ZhangBao, 2021 (26)	AUC-ROC	0.937	The scoring model demonstrates excellent performance in both discrimination (AUC) and calibration (H-L test).
	Sensitivity	87.9%	
	Specificity	90.1%	
	Hosmer-Lemeshow	0.844	
Laura Clarke, 2021 (27)	Precision	0.935/0.939	Both traditional scoring systems and machine learning decision trees demonstrated excellent and reliable performance, significantly outperforming other MRI criteria commonly used in clinical practice at the time.
	F-measure	0.935/0.939	
	AUC	0.882/0.874	
	Comparison with previous standards		
Jacqueline Palace, 2019 (30)	Rate Ratio	relapse rate	The study did not provide a single metric for the model’s overall discrimination or calibration. Its “performance” was primarily reflected in the high statistical significance of all identified risk factors and strong alignment between model-estimated risk values and clinical understanding.
	Hazard Ratio	Disability Incident	
Laura Cacciaguerra, 2019 (31)	Sensitivity	0.92/0.82	This diagnostic algorithm demonstrates high and consistent specificity, good sensitivity, and powerful differential diagnostic capability.
	Specificity	0.91/0.91	
	Accuracy	0.92/0.85	
	OR	38.62 (p<0.0001)	
Paula Barreras, 2018 (28)	Correct Classification Rate	From 67% to 87%	Incorporating clinical features such as the temporal progression of symptoms and motor examination significantly improves the model’s classification performance over conventional indicators (Gd+ and pleocytosis).
	AUC	From 0.32 to 0.67	
	Comprehensive Improvement Index	38%	
	Net Weight Categorization Improvement Index	34%	
Dean M.Wingerchuk, 2003 (29)	Sensitivity	82.4%	The model’s predictive power is statistically significant with adequate sensitivity and specificity; however, these performance metrics may be overestimated due to a lack of rigorous validation.
	Specificity	73.9%	
	Positive likelihood ratio	3.16	
	Hazard Ratio	Reported HR for each variable	

### Data availability

Of the 11 included studies, only one [Lucia Campetella et al. (35)], provided a model directly accessible to the public. The remaining studies did not offer prediction models that could be directly downloaded or used online. Among these, four studies had models whose construction details or scoring rules were fully published in their respective papers, but researchers, clinicians, or peers wishing to use these models must manually extract the information from the articles and implement them independently. There are no readily downloadable code or tools available, making the process highly cumbersome and error-prone, so these models are considered “theoretically applicable.” The other six studies presented models that are highly complex, lacking core parameters or code, rendering them usable only as research instruments and thus “unusable” (see **Table 5**).

**Table 5:** Evaluation of model usability and reproducibility in included studies.

Author, year	Fully disclosed (availability)	Acquisition method
Fan Yang, 2024 (32)	Unclean (Theoretically available)	The complete nomogram has been published.
Wenqin Luo, 2023 (33)	NO (Unavailable)	Scores must be calculated manually.
Lucia Campetella, 2023 (35)	YES (Available immediately)	Provided a comprehensive scoring system.
Liang Wang, 2022 (34)	Unclean (Theoretically available)	Provided key visualization tools for the model (Nomogram) and all predictor variables.
Laura Cacciaguerra, 2022 (52)	NO (Unavailable)	The dataset analyzed and the final algorithm are available on reasonable request.
Jingzi ZhangBao, 2021 (26)	Unclean (Theoretically available)	The complete scoring rules have been publicly released.
Laura Clarke, 2021 (27)	Unclean (Theoretically available)	The complete construction methodology and evaluation criteria have been publicly published.
Jacqueline Palace, 2019 (30)	NO (Unavailable)	Primarily detailed mathematical formulas, estimation procedures, and prediction algorithms.
Laura Cacciaguerra, 2019 (31)	NO (Unavailable)	MRI requires manual evaluation.
Paula Barreras, 2018 (28)	NO (Unavailable)	Manual integration of clinical/MRI/CSF features are required.
Dean M.Wingerchuk, 2003 (29)	NO (Unavailable)	Manual review of statistical parameters and comprehensive assessment are required.

In summary, while the majority of the studies have made valuable methodological contributions and pointing the way for future clinical practice, but their primary contribution lies in generating new medical knowledge rather than serving as direct diagnostic or treatment tools. From the perspective of clinical applicability, they have not yet become mature tools that are “truly ready for direct use by others.”

### Results of quality assessment

**Figure 2:**
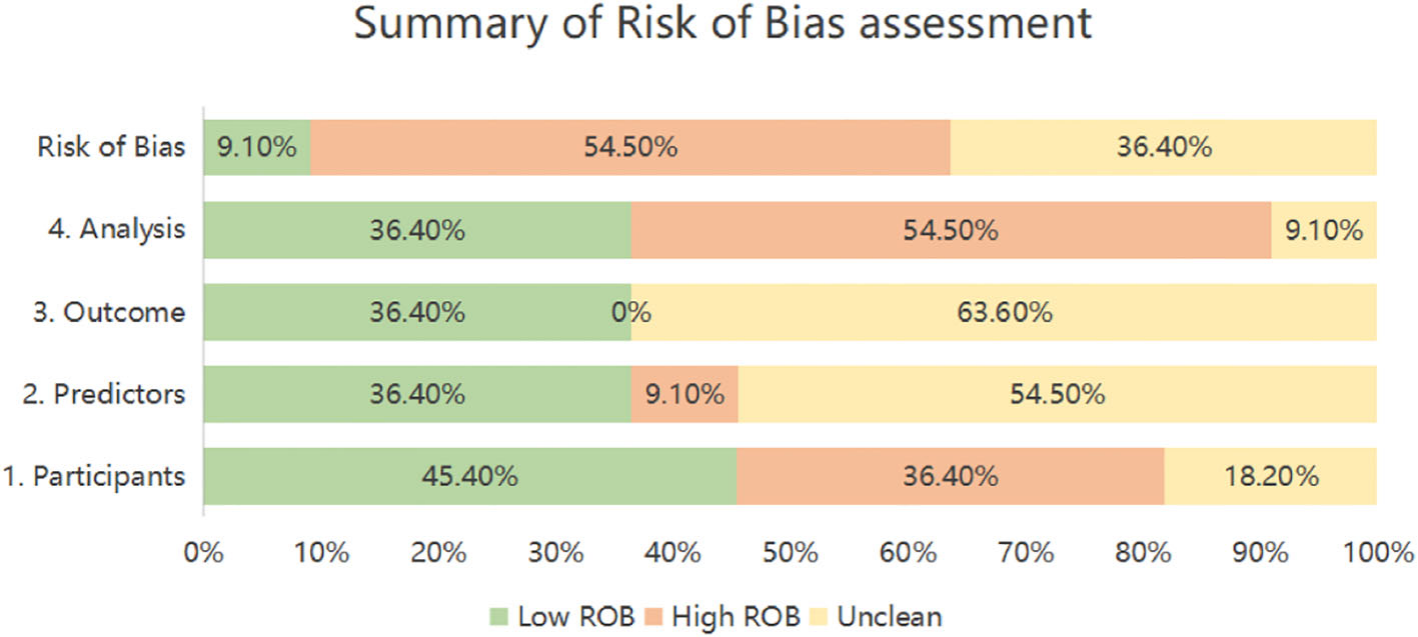
Summary of risk of bias assessment.

The PROBAST-based quality assessment revealed substantial methodological limitations. Only a single study (9.1%) exhibited low overall risk of bias. Conversely, six investigations (54.5%) demonstrated high bias risk, signaling deficiencies in development or validation methodology, while four studies (36.4%) presented unclear risk due to insufficient reporting transparency. Within the Participant domain, four studies (36.4%) incurred high bias risk principally because of inadequately specified inclusion/exclusion criteria (26–29), and two studies (18.2%) showed unclear risk owing to incomplete descriptions of data sources or participant selection methods (30, 31). Regarding Predictors, one study (9.1%) was rated high risk, potentially reflecting non-blinded assessment (28), while six studies (54.5%) held unclear risk as they omitted reporting quality control procedures for predictor measurement—a lapse potentially attributable to retrospective designs (26, 28–30, 32–34). For Outcomes, seven studies (63.6%) carried unclear risk, none having documented whether blinding was maintained between outcome and predictor evaluations (26, 28–30, 32–34). Analysis concerns were pronounced: six studies (54.5%) manifested high bias risk. Specific issues encompassed deficient handling of missing data (n=4 studies) (26, 28, 31, 35), exclusive use of apparent validation without further testing (n=5 studies) (27–29, 31, 35), problematic conversion of continuous variables to categorical formats (n=2 studies) (26, 27), and reliance on single-split internal validation alone (n=1 study) (31). An additional study (9.1%) exhibited unclear analytical risk due to unreported statistical details (30). (**Table 6**, **Figure 2**).

**Table 6:** PROBAST results of the included studies: summarizes the risk of bias and applicability in the included studies.

Author, year	Risk of bias				Applicability			Overall	
	1. Participants	2. Predictors	3. Outcome	4. Analysis	1. Participants	2. Predictors	3. Outcome	Risk of Bias	Applicability
Fan Yang, 2024 (32)	+	?	?	+	+	+	+	?	+
Wenqin Luo, 2023 (33)	+	?	?	+	+	-	+	?	-
Lucia Campetella, 2023 (35)	+	+	+	-	+	+	+	-	+
Liang Wang, 2022 (34)	+	?	?	+	+	+	+	?	+
Laura Cacciaguerra, 2022 (52)	+	+	+	+	+	+	+	+	+
Jingzi ZhangBao, 2021 (26)	-	?	?	-	+	+	+	-	+
Laura Clarke, 2021 (27)	-	+	+	-	+	+	+	-	+
Jacqueline Palace, 2019 (30)	?	?	?	?	+	+	+	?	+
Laura Cacciaguerra, 2019 (31)	?	+	+	-	+	+	+	-	+
Paula Barreras, 2018 (28)	-	-	?	-	+	+	+	-	+
Dean M.Wingerchuk, 2003 (29)	-	?	?	-	-	-	+	-	-

PROBAST, the Predictive Model Risk of Bias Assessment Tool; ROB, Risk of Bias.

Red indicates “high risk”; green indicates “low risk”; yellow indicates “unclear.”

+ indicates low ROB/low concern about applicability; - indicates high ROB/high concern about applicability; ? indicates unclear ROB/unclear concern about applicability.

**Figure 3:**
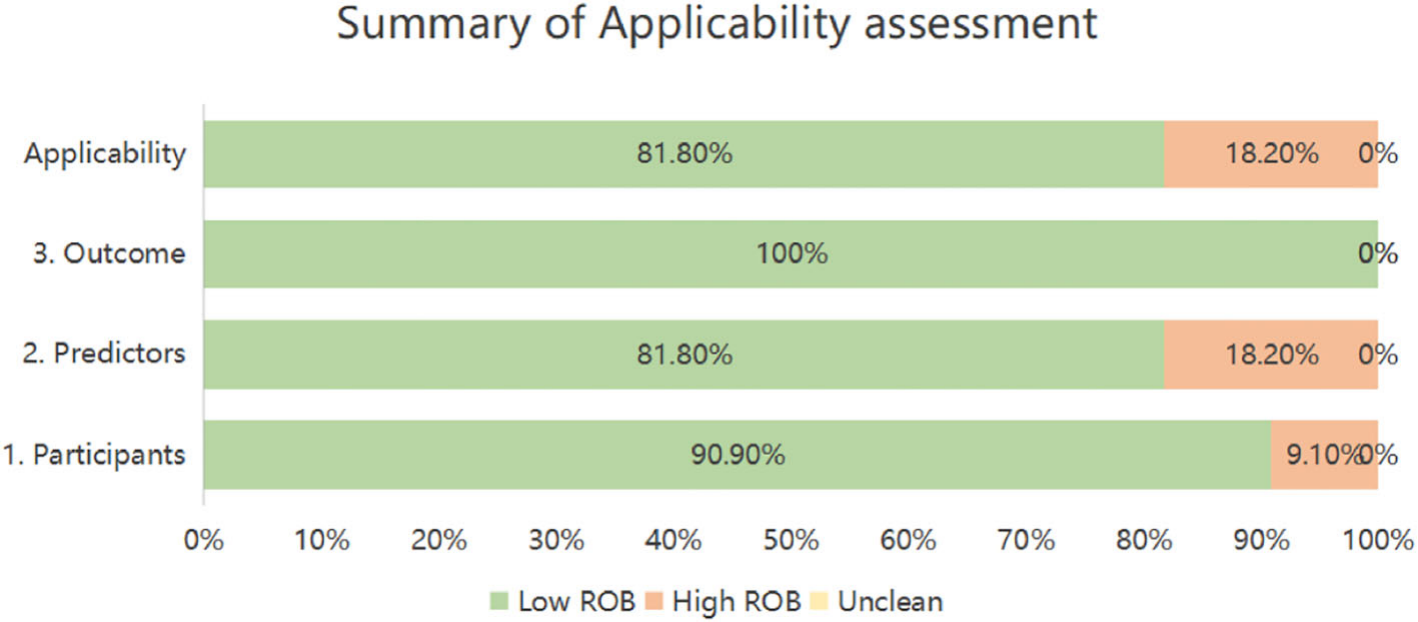
Summary of applicability assessment.

Applicability risk assessment revealed two studies (18.2%) with high overall risk, contrasting with nine (81.8%) demonstrating low applicability concerns. Domain-specific evaluation indicated high risk in Participants for one investigation (9.1%) due to notably limited sample size (29). Within Predictors, two studies (18.2%) received high-risk ratings stemming from suboptimal predictor measurement timing (29, 33). Conversely, all eleven studies exhibited universally low applicability risk in the Outcomes domain (**Table 6**, **Figure 3**).

### Assessment of comorbidity-related bias

Some of the included studies did not explicitly address comorbidity-related bias during the modeling process. These potential biases may lead to unmeasured confounding factors, limiting the accuracy and generalizability of the study results and models. The evaluation identified four studies with high bias, three with moderate bias, and four with low bias (see **Table 7**). Among these, studies with low bias clearly documented autoimmune or other underlying comorbidities and incorporated them into the analysis, demonstrating better bias control. Studies with moderate bias systematically excluded patients with severe comorbidities, leading to an underestimation of the negative impact of comorbidities on adverse outcomes, or presented risks of unmeasured comorbidity-related bias, or exhibited complex interactions between comorbidities and the disease under investigation. Studies with high bias either did not report or systematically adjust for comorbidities.

**Table 7:** Assessment of comorbidity-related bias.

Article	Degree of bias	Assessment criteria
Fan Yang, 2024 (32),	High	Common comorbidities were not excluded or analyzed as covariates.
Wenqin Luo, 2023 (33),	Middle	Systematically excluded patients with severe comorbidities, and underestimated the impact of complications.
Lucia Campetella, 2023 (35)	Low	\
Liang Wang, 2022 (34)	Low	\
Laura Cacciaguerra, 2022	Middle	There is a risk of unmeasured bias related to comorbidities.
Jingzi ZhangBao, 2021 (26)	Low	\
Laura Clarke, 2021 (27)	High	No intergroup comparisons or statistical adjustments were made for comorbidities.
Jacqueline Palace, 2019 (30)	High	Complications not mentioned or adjusted by the system.
Laura Cacciaguerra, 2019 (31)	High	No complications were reported or discussed.
Paula Barreras, 2018 (28)	Low	\
Dean M.Wingerchuk, 2003 (29)	Middle	Comorbidities and the study disease exhibit intertwined effects, making it impossible to isolate their independent effects.

### Meta-analysis of validation models included in the review

**Figure 4:**
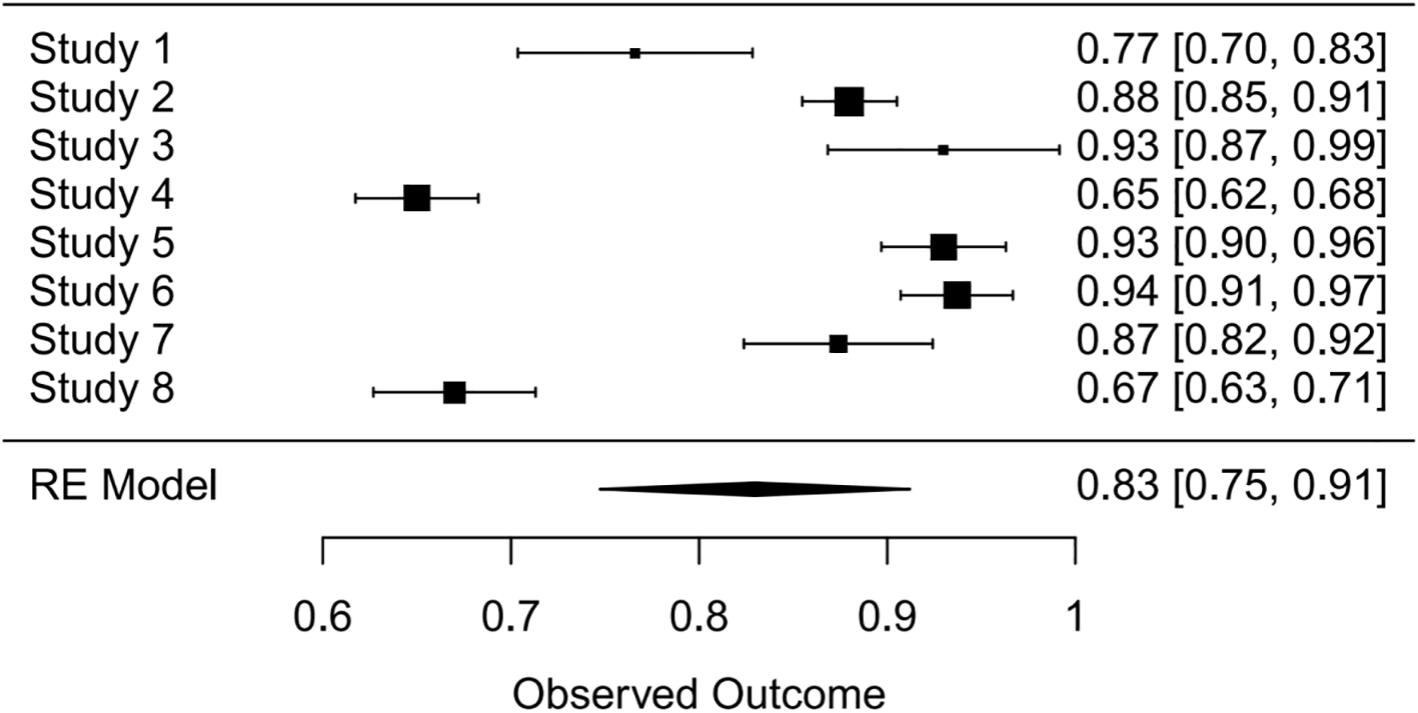
Forest plot visualizing summary AUC performance of validation models (n=8) under random-effects modeling.

**Figure 5:**
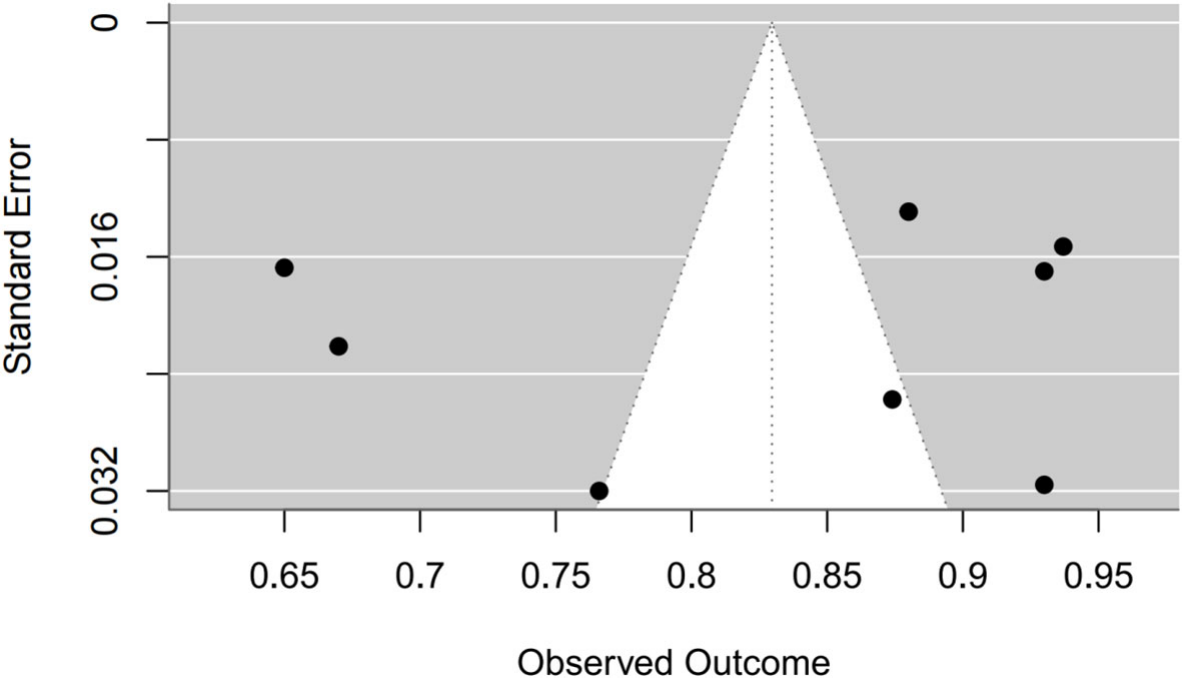
Funnel plot.

Synthesis of validation models proved feasible for eight studies (**Table 8**), as insufficient methodological reporting precluded inclusion of others. Although the investigation by Clarke et al. employed multiple modeling methodologies (27), all derived from a common cohort. Consequently, only objectively developed machine learning models underwent meta-analysis. A random-effects model yielded a pooled AUC of 0.83 (95% CI: 0.75–0.91). Substantial heterogeneity emerged (I² = 97.4%, p < 0.0001). Publication bias analysis via Egger’s test indicated non-significance (z ≈ -0.47; intercept ≈ 0.91, p = 0.656), corroborated by visual inspection of funnel and forest plots (**Figures 4**, **5**, **Supplementary Figures 1–2**).

**Table 8:** A detailed list of the specific AUC values of the predictive models included in the system overview.

Study	Article	AUC	n	SE	vi
1	F. Yang (2024) (32)	0.766	177	0.03182260	0.0010126780
2	W. Luo (2023) (33)	0.88	640	0.01284523	0.0001650000
3	L. Campetella (2023) (35)	0.93	66	0.03140643	0.0009863636
4	L. Wang (2022) (34)	0.65	820	0.01665650	0.0002774390
5	L. Cacciaguerra (2022) (52)	0.93	228	0.01689752	0.0002855263
6	J. ZhangBao (2021) (26)	0.937	255	0.01521493	0.0002314941
7	L. Clarke (2021) (27)	0.874	168	0.02560273	0.0006555000
8	P. Barreras (2018) (28)	0.67	457	0.02199562	0.0004838074
9	J. Palace (2019) (30)	Unknown	441	/	/
10	L. Cacciaguerra (2019) (31)	Unknown	181	/	/
11	D. M. Wingerchuk (2003) (29)	Unknown	80	/	/

AUC, Area Under the Curve. Diagnostic performance was interpreted using established AUC thresholds: 0.5–0.7 (poor), 0.7–0.8 (moderate), 0.8–0.9 (good), and 0.9–1.0 (excellent) discrimination.

## Discussion

Myelitis is a broad concept encompassing various subtypes, making the clarity of myelitis diagnosis particularly important (4). Among numerous diagnostic indicators, MOG-related antibodies (such as IgG) (36, 37) and AQP4-related antibodies (such as IgG) (38, 39) play a central role in the diagnosis of myelitis. Preliminary statistics indicate that there is currently a wealth of research related to poliomyelitis and NMOSD, likely due to the prevalence and severity of these two diseases. The clinical symptoms of myelitis are largely similar, primarily manifesting as a series of symptoms following nervous system injury. Common clinical treatments include antiviral therapy, steroids, immunosuppressants, and plasma exchange (40–42), but these treatments are associated with certain adverse reactions and side effects (43). Therefore, early identification of effective clinical biomarkers, risk factor assessment, and the establishment of predictive models are strategies of significant clinical importance for predicting disease occurrence and progression, prognosis, recurrence, and monitoring treatment response. These strategies facilitate early identification and intervention, thereby significantly reducing adverse prognoses. This systematic review identifies a growing proliferation of myelitis prediction models, predominantly derived from single-center datasets. Among 11 evaluated models, internal/external validation demonstrated moderate-to-good predictive utility (AUC range: 0.65–0.937). However, PROBAST assessment revealed significant methodological limitations: six studies carried high bias risk and four exhibited unclear risk, substantially constraining clinical applicability. Meta-analysis of eight validated models produced an aggregate AUC of 0.83 (95% CI: 0.75–0.91), though extreme heterogeneity (I²=97.4%) was observed—likely attributable to population, predictor, and methodological variations. Further compromising reliability, inconsistent adherence to the TRIPOD reporting guidelines was noted across studies, introducing transparency deficits and potential bias (44, 45). Consequently, advancing this field necessitates: (1) development of models with expanded cohorts; (2) methodologically rigorous designs; (3) multicenter external validation; and (4) stringent reporting transparency.

Valuable insights can be gleaned from the model development processes of the included studies: Only three studies conducted both internal and external validation, while five studies only performed internal validation, despite the utilization of multicenter data in some cases. For example, leveraging multicenter data, the study by Cacciaguerra (31) et al. omitted rigorous external validation. Additionally, while random split validation represents an internal validation technique, it fails to mitigate model overfitting concerns (46). Wenqin Luo et al. employed LASSO regression and multivariate Cox regression for data analysis, demonstrating commendable handling of the models, whereas other models often overlooked this aspect. However, there were some predictors that involved variables which could not be obtained in a timely manner. Although Liang Wang et al. also used an extended Cox regression model and conducted both internal and external validation, the study was retrospective and failed to report the use of blinding, resulting in an unclear risk of bias. During the model development process, Laura Clarke et al. utilized both machine learning and scoring algorithms. Some studies indicate that compared to traditional logistic regression, machine learning methods often produce more objective and higher accuracy results (47). Machine learning approaches offer solutions to common methodological challenges—including limited samples, continuous variable handling, and predictor selection—encountered in conventional modeling. However, these techniques introduce new limitations: inadequate visualization frameworks and persistent reporting transparency deficits. The current review further identified substantial undisclosed analytical details. Consequently, methodological selection should be evidence-based and context-driven. While all evaluated models demonstrated moderate-to-high discriminative capacity, significant bias risks remain. Critical improvement areas include: (1) strengthening data provenance (prospective/retrospective; cohort/case-control designs); (2) standardizing predictor-outcome temporal alignment; (3) implementing blinding protocols; (4) ensuring adequate event rates; (5) optimizing continuous variable treatment; (6) refining predictor selection; (7) addressing data complexity; and (8) enhancing calibration and fitting procedures.

The incorporated prediction models demonstrate translational relevance through clinically informative predictors. Notably, gender emerged as a recurrent predictor across six studies, aligning with established epidemiological evidence of heightened myelitis incidence in females versus males (48, 49). Nevertheless, variations in age-sex distributions across cohorts challenge the universal generalizability of these predictors. For instance, two of the included studies reported a slightly higher risk in males (26, 33). Secondly, age is an important indicator commonly used in predicting the prognosis of myelitis, as it is associated with numerous diseases. The age of onset spans the entire age range, encompassing both pediatric and elderly populations. Some studies have shown a close relationship between older age at onset and poorer prognosis of myelitis (50). For example, Romina Mariano et al. (49) indicated that patients aged 50 and above exhibit higher levels of disability post-onset, whereas patients under 50 have higher recurrence rates after onset. Aditya Banerjee et al. (51) reported that patients under 35 years old may have better prognoses after recurrence compared to those over 35. Therefore, early detection, diagnosis, and intervention strategies should be adopted for younger populations, while increased attention and care are necessary for older populations. Additionally, the number of relapses, central spinal lesions, and longitudinally extensive transverse myelitis (LETM) are also commonly used predictors. The number of relapses is often used to predict the risk and prognosis of myelitis, the higher the number of relapses, the greater the risk of adverse outcomes and poorer prognosis (29, 33, 34). Imaging features such as central spinal lesions and LETM are frequently used in the classification and diagnosis of myelitis. Moreover, predictors identified across the 11 models constitute valuable candidates for future diagnostic, prognostic, and risk-modeling research. While urgent clinical implementation of myelitis prediction tools remains imperative, rigorous validation trials are essential to confirm their efficacy in: (1) enhancing early diagnostic accuracy, and (2) mitigating recurrence/progression risks. Successful validation will enable deployment across broader populations, ultimately improving patient prognoses and rehabilitation outcomes.

However, it is noteworthy that there is often an inverse relationship between model complexity and its clinical usability. Although these studies have yielded significant scientific discoveries, most of their models lack immediate accessibility, which substantially limits their dissemination, validation, and application in clinical practice and subsequent research. Therefore, enhancing model transparency and accessibility is of paramount importance to advance the field from “publishing papers” to “serving clinical needs”.

## Limitations

Several limitations warrant consideration. First, predominant single-center derivation of included studies potentially constrains generalizability across institutions and regions. Application in diverse settings may thus require population-specific recalibration, underscoring the need for future models developed in multi-ethnic cohorts. Second, methodological and reporting heterogeneity permitted meta-analysis of only eight validation models from distinct studies. This restricted synthesis precluded comprehensive exploration of heterogeneity sources and compromised statistical power in publication bias assessment. Importantly, these constraints do not invalidate model evaluations but directly mirror the methodological gaps identified elsewhere in our analysis. Third, given that some models are not directly accessible, our conclusions regarding their clinical applicability are primarily based on performance metrics reported in the literature. Future prospective validation in cohorts with available models is necessary to further corroborate these findings. Forth, language restrictions excluded non-English publications, potentially omitting relevant research.

## Conclusion

This systematic review synthesizes 11 models derived from 11 studies. Meta-analysis of eight validated models demonstrated good discriminative capacity (pooled AUC 0.83; 95% CI: 0.75–0.91). However, PROBAST assessment revealed substantial methodological concerns: six studies carried high risk of bias, four exhibited unclear risk, and two raised applicability issues. Additionally, we observed that the current majority of myelitis prediction models have limited open accessibility, which may restrict their independent validation in external cohorts and widespread adoption in clinical practice. Current reporting practices often fall short of PROBAST standards, underscoring the urgent need for researchers to rigorously employ PROBAST checklists and adhere to TRIPOD reporting guidelines. Furthermore, we encourage the provision of complete model coefficients in published papers or the sharing of code via open-source platforms to enhance research transparency and reproducibility. Future model development must prioritize multicenter designs, larger cohorts, methodologically robust validation, and enhanced transparency.

## Data Availability

The original contributions presented in the study are included in the article/**Supplementary Material**. Further inquiries can be directed to the corresponding author.
