# Supplementary figures and images for: Exploratory analysis of predictive models in the field of myelitis: a systematic review and meta-analysis
Source: Front Immunol. 2025 Oct 2;16:1669338. doi: 10.3389/fimmu.2025.1669338 (PMC12527870; doi:10.3389/fimmu.2025.1669338)

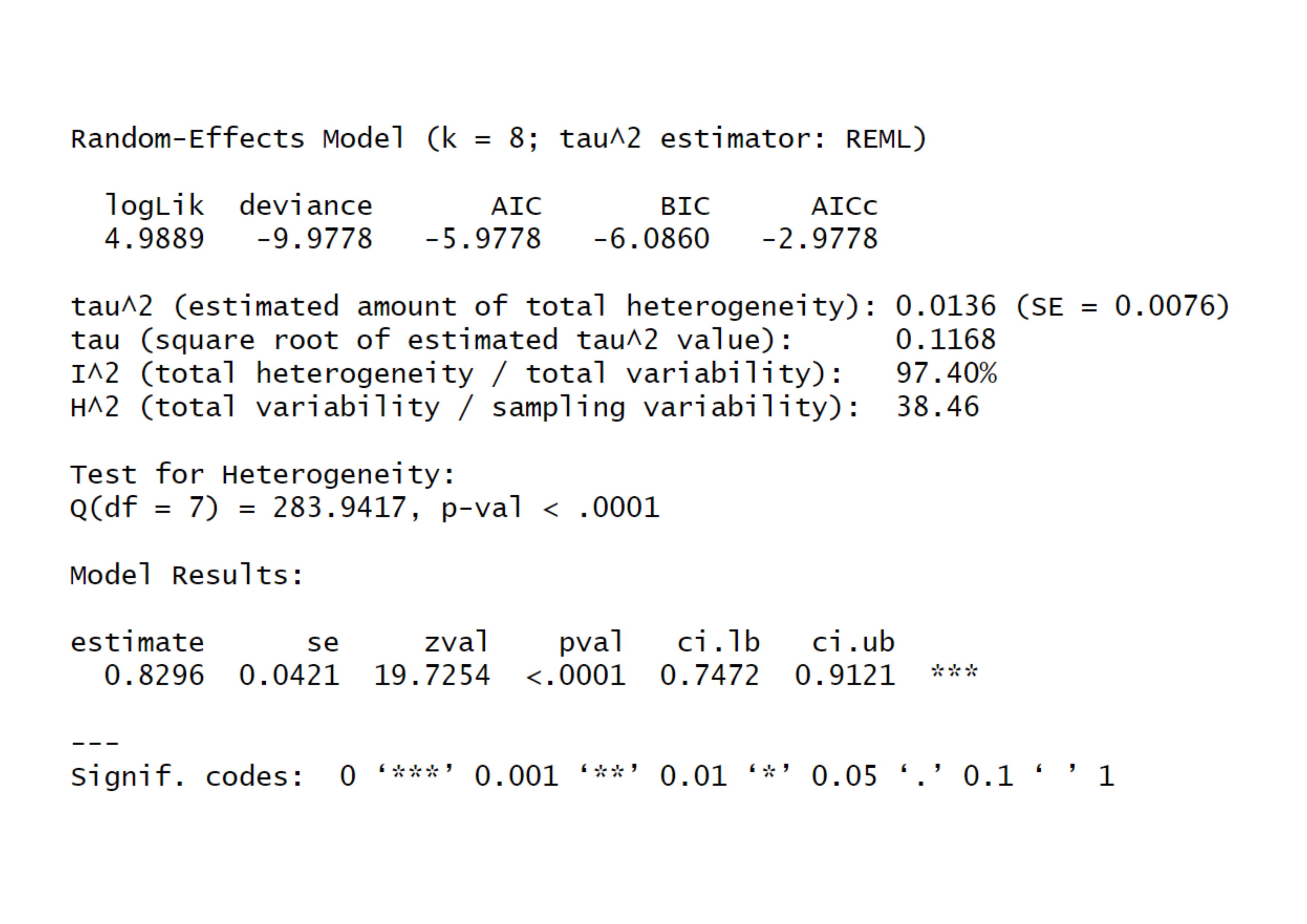

Supplement: Supplementary file 2 [file Image1.jpeg]

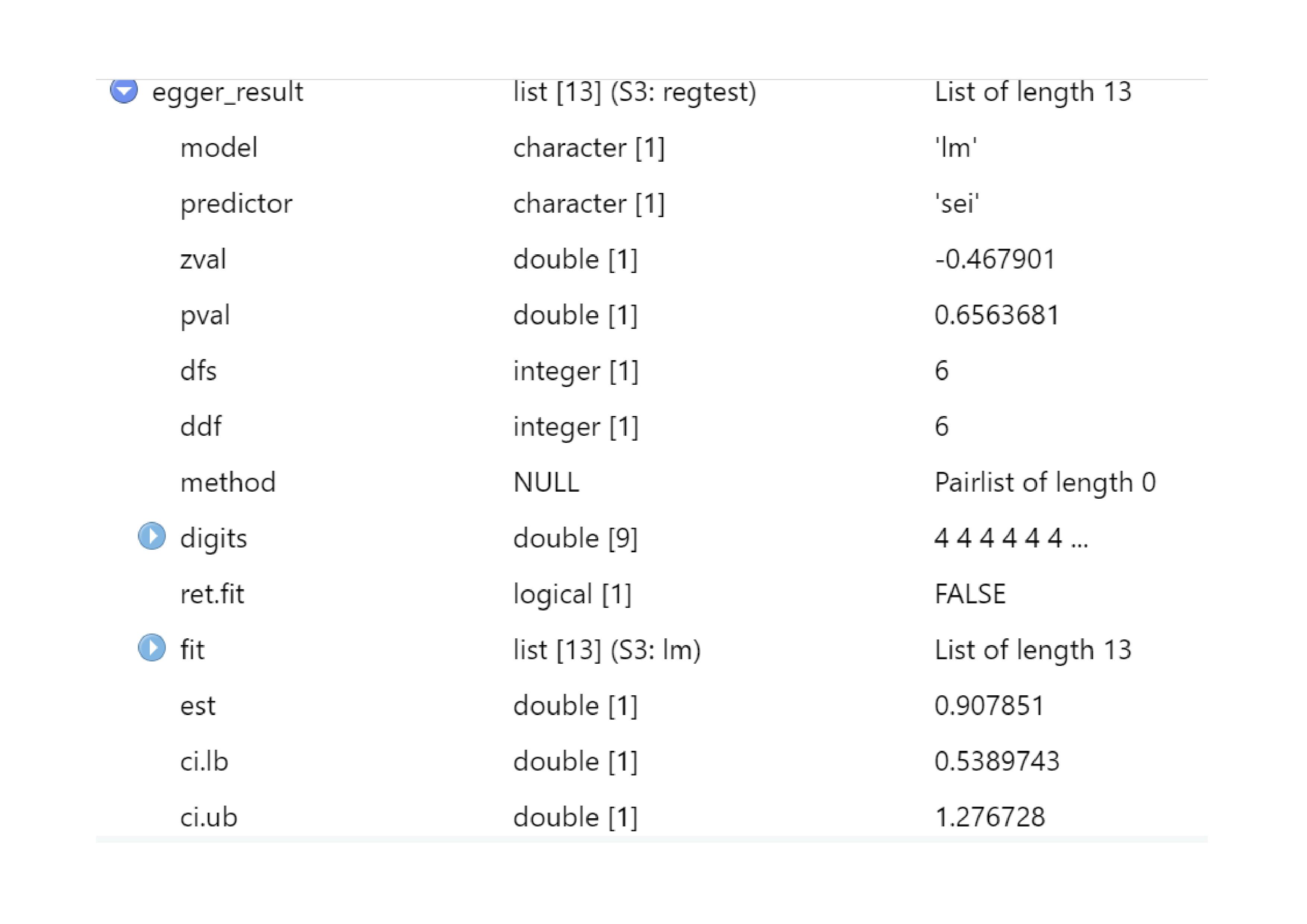

Supplement: Supplementary file 3 [file Image2.jpeg]
